# Acupuncture Alleviates Corneal Inflammation in New Zealand White Rabbits with Dry Eye Diseases by Regulating *α*7nAChR and NF-*κ*B Signaling Pathway

**DOI:** 10.1155/2022/6613144

**Published:** 2022-11-14

**Authors:** Ning Ding, Qingbo Wei, Qian Xu, Chengyong Liu, Yongcheng Ni, Jun Zhao, Wanli Xu, Weiping Gao

**Affiliations:** ^1^Department of Nutrition, Nanjing University Of Chinese Medicine, Nanjing City, Jiangsu Province 210023, China; ^2^Nanjing University of Chinese Medicine, Nanjing City, Jiangsu Province 210023, China; ^3^Affiliated Hospital of Nanjing University of Chinese Medicine, Nanjing City, Jiangsu Province 210029, China; ^4^Jiangsu Province Hospital, Nanjing City, Jiangsu Province 210029, China

## Abstract

**Purpose:**

The purpose of this study is to determine the mechanism of improvement in dry eye diseases (DEDs) treated by acupuncture. The inflammatory molecules and related pathways will be analyzed in our study.

**Methods:**

In order to establish the animal model for DEDs, healthy New Zealand white rabbits were treated with scopolamine (Scop) hydrobromide for 21 consecutive days. After 21 days, acupuncture, fluorometholone (Flu), and *α*7nAChR antagonist (*α*-BGT) treatments were performed, and the Scop injections were continued until day 35. The therapeutic effect of acupuncture on DED inflammation was evaluated by corneal fluorescence staining, tear film rupture time, tear flow measurement, in vivo confocal microscopy (IVCM), corneal histopathology, and cytokine protein chip technology. The influence of acupuncture on the corneal pathology and inflammatory factors ACh, *α*7nAChR, and NF-*κ*B was detected by enzyme-linked immunosorbent assay (ELISA) and western blot.

**Results:**

Compared with the group Scop, acupuncture can significantly reduce corneal staining and increase the tear film rupture time and tear flow, which are accompanied by a decrease in corneal epithelial detachment and lymphocyte infiltration. Acupuncture can relieve the inflammation of corneal stroma and mitigate the expression of proinflammatory factors and chemokines. Acupuncture can upregulate the expression of ACh and *α*7nAChR and downregulate the expression of NF-*κ*B.

**Conclusion:**

Our findings demonstrate that acupuncture can alleviate corneal inflammation in New Zealand white rabbits with DEDs via *α*7nAChR and NF-*κ*B signaling pathway regulation. The expression indicates that *α*7nAChR/NF-*κ*B signaling pathway may be active and that acupuncture is a potential therapeutic target for dry eye.

## 1. Introduction

Dry eye diseases (DEDs) are multifactor ocular surface diseases, characterized by unbalanced tear film homeostasis and accompanied with eye discomfort. The main pathophysiological mechanisms of DEDs include unstable tear film, increased tear osmotic pressure, ocular surface inflammatory response, and neurological abnormalities [1]. As DEDs develop, the life quality of patients with DEDs gradually declines, with some even experiencing psychological illnesses such as depression and anxiety. Inflammation has been identified as the key mechanism of the physiological and pathological pathogenesis of DEDs [2]. The excessive production of proinflammatory cytokines and chemokines causes the disruption of physiological homeostasis [3], leading the DEDs to progress. Therefore, inflammatory suppression in the ocular surface is the key point in the treatment of DEDs. It is reported that DEDs are closely related to the central nervous system [4]. Previous studies have also shown that the cholinergic anti-inflammatory pathway (CAP) can stimulate inflammatory molecular signaling by integrating the afferent vagus nerve into the central nervous system. Moreover, efferent nerve endings are near the immune cells of acetylcholine (ACh) and release into the reticular endothelial tissue, which can bind with *α*7nAChR receptors on the surface of macrophages to transmit vagal signals. CAP plays an important anti-inflammatory role by regulating the production of cytokines in cells, which is mainly related to the NF-*κ*B signaling pathway [5, 6].

It is demonstrated that acupuncture has a certain effect in the treatment of DEDs [7], which is to prolong the tear film rupture time and reduce conjunctival inflammation [8, 9]. Some published studies have shown that acupuncture can change the morphology of lacrimal glands in rabbits with DEDs and increase the secretion of tears; however, no mechanistic explanation has been given [10]. Several recent studies have shown that acupuncture can increase the expression of *α*7nAChR while attenuating tissue inflammation, further reducing tissue damage [11–14]. In this study, systemic administration of the muscarinic cholinergic inhibitor scopolamine hydrobromide could inhibit tear secretion in New Zealand rabbits to mimic DEDs [15]. We evaluated the treatment efficacy of acupuncture on DED manifestations in the experimental DEDs rabbit model, including tear secretion, ocular surface stability, and inflammatory markers. The aim of this research is to explore whether acupuncture is able to inhibit the corneal inflammation associated with DEDs by regulating *α*7nAChR, which could subsequently reduce the inflammatory response caused by the NF-*κ*B pathway activation.

## 2. Materials and Methods

### 2.1. Experimental Animals

Healthy New Zealand rabbits (male and female, 2-3 months old, weighing about 1.5 kg) were purchased from Qinglong Mountain Experimental Animal Farm (Nanjing, China) and raised in the pharmacology laboratory of Jiangsu Provincial Hospital of Traditional Chinese Medicine. The experimental animals were housed in ambient conditions of room temperature (22 ± 2°C), at a relative humidity of 60% ± 5%, with an alternating 12-hourlight-dark cycle, and with water and standard feed provided constantly available. The experimental protocol was approved by the Institutional Animal Care and Use Committee of Nanjing University of Chinese Medicine (Approval ID: 201809A018).

### 2.2. Instruments and Reagents

The following instruments and reagents were used in this study: Scopolamine hydrobromide (Chengdu Pufeide Biotech Co., Ltd., JOT-10515, China); fluorometholone eye drops (0.1%, Shentian Pharma, J20180068, China); *α*7nAChR antagonist *α*-BGT (promoter, Catalog No:pk-ca707-00010-1, Germany); tear detection filter paper strip (Tianjin Jinmin New Technology Development Co., Ltd., China); IVCM (HRT III RCM, Heidelberg Company, Germany); obuvacaine hydrochloride eye drops (Ginseng Pharmaceutical Co., Ltd., China); Vidisci gel (0.2% Bausch and Lomb, China); RM2135 slicer (LEICA, Germany); DMLS2 optical microscope (LEICA, Germany); Qal-CYT-1 kit (RayBiotech, Inc., Norcross, GA, USA); InnoScan 300 Microarray Scanner (Innopsys, France); Ach ELISA kit (Nanjing Jinyiba Biotechnology Co., Ltd., Catalog No:JEB14612, China), *α*7nAChR ELISA kit (Nanjing Jinyiba Biotechnology Co., Ltd., Catalog No:JEB14612, China); enzyme-labeled instrument (Bio-Tek ELx800; Bio-tek Instruments, USA); phospho-NF-*κ*B P65 (1 : 1000; catalog No:3033S; Description Cell Signaling Technology); NF-*κ*B p65 (1 : :1000; catalog No: 08101524a, ENZO); GAPDH (1 : :1000; Catalog No:AB8245, Abcam); hrP-conjugated goat anti-rabbit IgG (1 : 500; Catalog No:FMS-RB01, FcMACS); Imager (ChemiDoc XRS System; BioRad Laboratories, Japan).

### 2.3. Experimental Procedures

**Figure 1:**
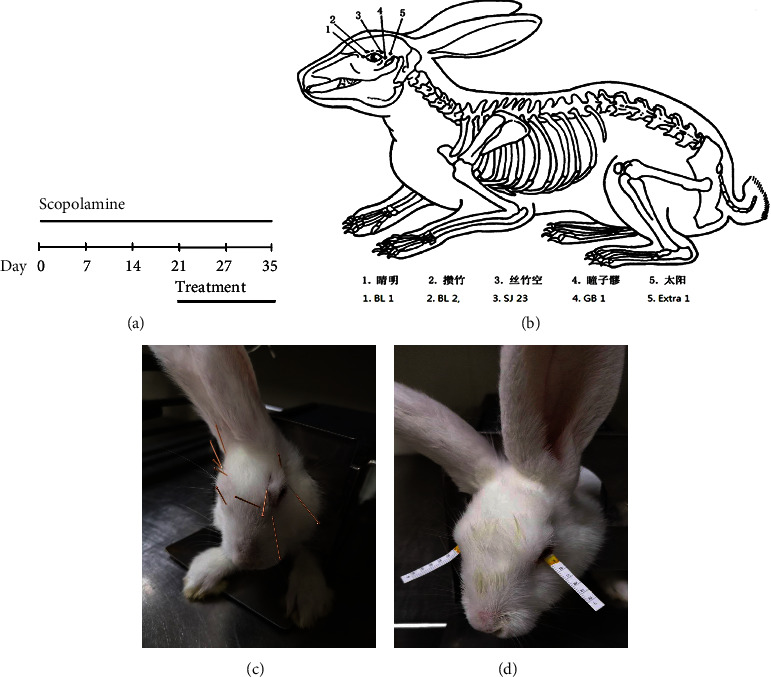
(a) Experimental design for acupuncture treatment and (b) the locations of acupuncture points. (c) Acupuncture operation and (d) the Schirmer I test.

Before the experiment, all animals were required to have no abnormality in the anterior segment of the eye, and the tear flow was required to be greater than 10 mm/5 min. In our first experiment, 30 healthy adult New Zealand white rabbits were randomly divided into the following five groups: group control (Con), group scopolamine (Scop), group sham acupuncture (Scop + Sham), group acupuncture (Scop + Acup), and group fluorometholone (Scop + Flu). Group Con received no treatment, the other five groups were given 2.0 mg/mL of scopolamine hydrobromide, and injected subcutaneously four times a day (8 : 00, 11 : 00, 14 : 00, and 18 : 00) for 35 days. Treatment began after the DEDs model was completed, on the 22nd day (Figure 1(a)). Group Scop + Sham: sham acupuncture treatment (Jingming BL1, Cuanzhu BL2, Sizhukong SJ23, Taiyang Ex-HN5, and Tongziliao GB1) was initiated, with a blunt needle pointing at the acupoints but not inserting into the skin, once a day for 14 consecutive days. Group Scop + Acup: the acupuncture point was the same as that of group Scop + Sham. The needle was retained for 15 minutes, once a day, for 14 days (Figures 1(b) and 1(c)). Group Scop + Flu : fluorometholone eye drops were administered three times a day (8 : 00, 13 : 00, and 18 : 00) for 14 days after successful modeling. The tear amount (Schirmer I test, SIt), fluorescein staining score (FL), and tear break-up time (BUT) were measured at 1, 7, 14, 21, 28, and 35 days, and the experimental animals were euthanized by air embolism after confocal microscope examination on day 35.

In the second experiment, 30 healthy adult New Zealand white rabbits were randomly divided into five groups: Scop, Scop + Acup, Scop + *α*-BGT, Scop + *α*-BGT + Flu, and Scop + *α*-BGT + Acup. The specific *α*7nAChR antagonist *α*-BGT was injected into the ear vein from day 22, 4.0 *μ*g/kg daily until the animals were euthanized by air embolism on day 35.

### 2.4. Schirmer I Test (SIt)

A tear detection filter paper strip was folded at one end and put into the conjunctival sac of the outer third of the rabbit's lower eyelid. After 5 minutes, the filter paper was taken out and the wetting length was measured from the folding point (Figure 1(d)).

### 2.5. Corneal Fluorescein Staining Score (FL)

The corneal staining filter paper strip was put into the lower eyelid fornix of the rabbit, the staining filter paper strip was wetted, and the fluorescein was rapidly and evenly distributed on the cornea through the eye blink. The corneal epithelial injury was graded with a cobalt blue filter. The cornea was classified into four quadrants and the scores were made, respectively. The modifications were scored between 0 and 3 points as follows: 0 points if absent, 1 point if fewer than five spots, 2 points if more than five spots, and 3 points if a large area of fluorescein plaque was evident. Finally, the score of each grade was added up, giving a total potential score of 12 [16].

### 2.6. Tear Break-Up Time (BUT)

The time from the corneal fluorescence staining to the appearance of the first corneal dry spot was measured. The testing time was recorded under a slit lamp, and the BUT test was repeated three times for each rabbit.

### 2.7. In Vivo Confocal Microscopy (IVCM)

The rabbit's head was fixed in a corneal laser confocal microscope, and obuvacaine hydrochloride eye drops were used for topical anesthesia. After setting the blepharostat, transparent Vidisci gel was applied on the surface of a 40-foldwater-immersed conical objective lens. The lens was moved forward slowly, and the recording button was pressed when the corneal cells were visible and the image was clearly in the center of the display screen. Photographs of the stroma layer were taken in the center of the cornea in the microscopic field.

### 2.8. Optical Microscope

After euthanasia, corneas were collected and fixed in 4% paraformaldehyde for 24 hours. The corneal epithelial specimens were separated without contact with the epithelium, and the corneal size was 2 × 2 mm. Then, the dissection, paraffin embedding, RM2135 slicing, H&E staining, and DMLS2 optical microscope observation were performed.

### 2.9. Cytokine Quantification Array

Rabbit cytokine quantification arrays were performed using the Qal-CYT-1 kit. All protein was extracted from the cornea using the tissue protein extraction kit, and protein concentration was determined by the BCA method (Pierce, No. 23227). According to the instructions of the manufacturer's kit, the expressions of 10 cytokines in six groups of corneas (including IL-1A, IL-1B, IL-8, IL-17A, IL-21, leptin, MIP-1B, MMP-9, NCAM-1, and TNF-a) were detected by rabbit cytokine quantification array and repeated four times. The InnoScan 300 Microarray Scanner was applied to scan signals using Cy3 excitation curves.

### 2.10. ELISA

The cornea of the rabbit was immediately taken out after euthanasia. After rinsing with normal saline, the cornea was fully homogenized in an ice bath and diluted with 300 *μ* salines. After centrifugation, the supernatant was taken and stored at −80°C for further experiments. The ACh and *α*7nAChR were detected using the ELISA kit, and an enzyme-labeled instrument was used for the determination of ACh and *α*7nAChR.

### 2.11. Western Blot

RIPA lysis buffer was used to extract corneal proteins from each group. The supernatant was centrifuged, the total protein concentration was determined by the BCA method, and the supernatant was calculated. Electrophoresis separation was carried out in SDS-PAGE gel with quantified protein samples. After electrophoresis, the gel and the membrane were cut into small strips according to the molecular weight of the protein. After the membrane was completed, 5% milk was blocked for 1 h, then the membrane was incubated in phospho-NF-*κ*B P65, NF-*κ*B p65, and GAPDH at 4°C overnight. The next day, it was washed three times with 0.05% Tween-20 Tris buffer saline for 10 minutes each time, then incubated in the hrP-conjugated goat anti-rabbit IgG for 1 hour. The membrane was washed in TBST three times, for 10 minutes each time. The membrane was covered by ECL liquid, then detected by using an Imager.

## 3. Statistical Analyses

Data were represented as mean ± SEM. Statistical significance was evaluated by two-way ANOVA with Bofferroni's post hoc test or the Mann–Whitney test using graphing software (GraphPad Prism 8.0; GraphPad Software, San Diego, CA, USA). *P* < 0.05 was considered statistically significant.

## 4. Results

### 4.1. Acupuncture Treatment Can Increase Tear Flow and BUT

**Figure 2:**
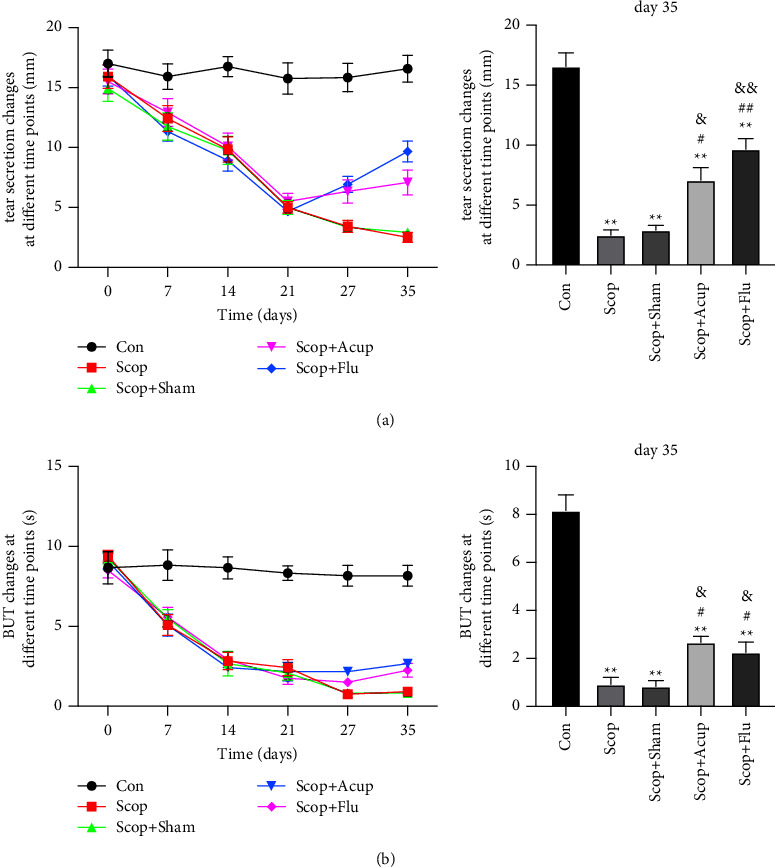
The effect of acupuncture treatment on tear flow and tear film rupture time in DED induced by scopolamine hydrobromide. (a) Tear fluid flow; (b) tear film rupture time. Quantitative data are shown as mean ± SEM. ^*∗∗*^*P* < 0.01 vs group Con; ^#^*P* < 0.05, ^##^*P* < 0.01 vs group Scop; ^&^*P* < 0.05, ^&&^*P* < 0.01 vs group Scop + Sham.

Compared with the group Con, the SIt of the group Scop and the group Scop + Sham decreased significantly and had a continuous reduction from day 14. After 21 days of treatment, compared with the group Scop and the group Scop + Sham, SIT in the group Scop + Acup significantly increased (Day 35:7.1 ± 3.6 vs. 2.9 ± 1.2 mm, *P* < 0.05; 7.1 ± 3.6 vs. 2.9 ± 1.2 mm, *P* < 0.05, Figure 2(a)). Compared with the group Scop and the group Scop + Sham, tear flow in the group Scop + Flu also significantly increased (*P* < 0.01, Figure 2(a)).

Compared with the group Con, the BUT of the group Scop and the group Scop + Sham was decreased 14 days (*P* < 0.05, Figure 2(b)). Compared with the group Scop and the group Scop + Sham, the BUT in the group Scop + Acup was increased (Day 35:2.7 ± 0.9 vs. 0.9 ± 1.0 s; 2.5 ± 0.9 vs. 0.8 ± 0.8 s, *P* < 0.05, Figure 2(b)). Compared with the group Scop and the group Scop + Sham, the BUT in the group Scop + Flu was also increased (*P* < 0.05).

### 4.2. Acupuncture Treatment Can Reduce Corneal Epithelial Damage

**Figure 3:**
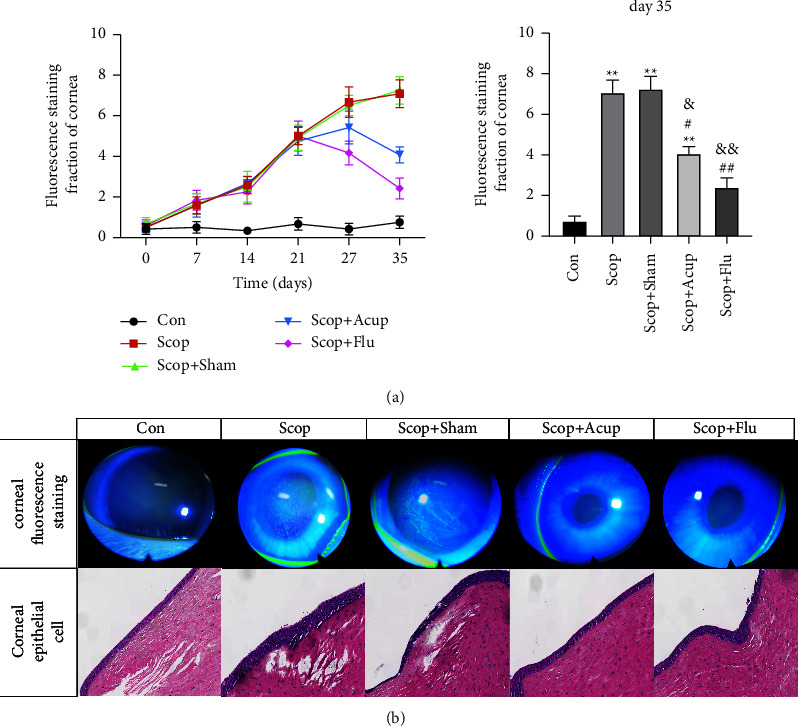
The effect of acupuncture treatment on corneal fluorescence staining and the histopathological images of the cornea (hematoxylin-eosin staining, ×10 times) on the 35th day in DED induced by scopolamine hydrobromide. (a) Corneal fluorescence staining score and (b) corneal fluorescence staining and the histopathological images of the cornea. Quantitative data are expressed as mean ± SEM. ^*∗*^*P* < 0.05, ^*∗∗*^*P* < 0.01 vs group Con. ^#^*P* < 0.05, ^##^*P* < 0.01 vs group Scop; ^&^*P* < 0.05, ^&&^*P* < 0.01 vs group Scop + Sham.

Compared with the group Scop and the group Scop + Sham, the FL score was significantly reduced in the group Scop + Acup on day 35 (4.1 ± 1.4 vs 7.1 ± 2.4 points, 4.1 ± 1.4 vs 7.3 ± 2.3 points, *P* < 0.05, Figure 3(a)). Meanwhile, compared with the group Scop and the group Scop + Sham, FL of the group Scop + Flu also significantly reduced the FL (*P* < 0.01, Figure 3(a)). The corneal fluorescence staining on the 35th day is shown in Figure 3(b). The corneal epithelium of the rabbits in the group Con had almost no staining, whereas those in the group Scop and the group Scop + Sham were significantly stained. After treatment, the ocular surface staining of the group Scop + Acup and the group Scop + Flu decreased, and the staining was scattered as dots.

H&E staining results showed that hyperkeratotic squamous epithelial cells, lymphocyte infiltration, increasing focal epithelial cell layers, and shedding surface epithelial cells were seen on the surface of corneal tissues in the group Scop and the group Scop + Sham. Compared with these groups, the shedding corneal epithelial and lymphocyte infiltration decreased in the group Scop + Acup, and the group Scop + Sham (Figure 3(b)).

### 4.3. Acupuncture Treatment Improves Corneal Stromal Inflammation

**Figure 4:**
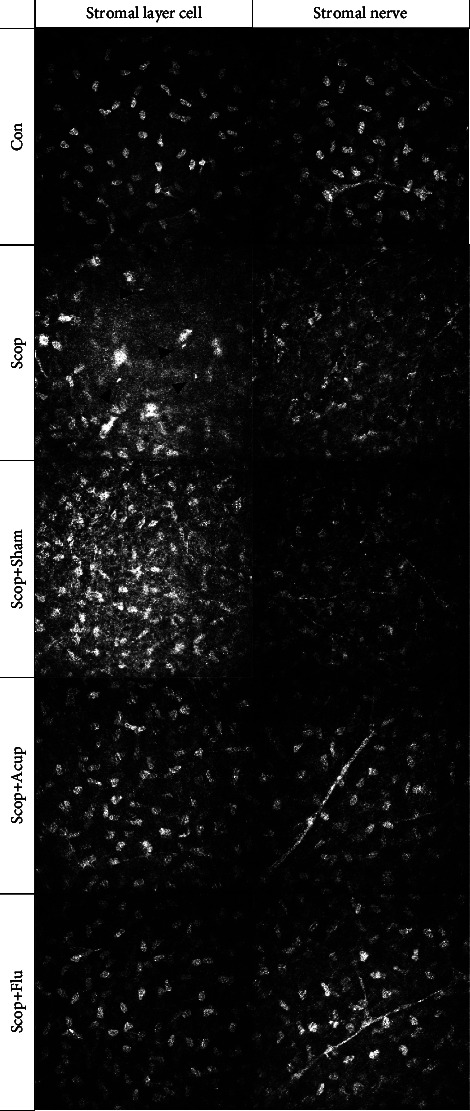
The IVCM images of the anterior and posterior stromal layers under the cornea on the 35th day. Quiet stromal cells, a small number of globular immune cells, and nerve fibers (Con). A large number of globular immune cells as shown by the arrow and activated stromal layer cells with unclear borders and irregular sizes. Areas with irregular intercellular spaces were seen. A change in nerve width was seen with the decreasing nerve branch reflectivity (Scop). Disordered stromal cells and intermittently thinner nerve fibers with high tortuous (Scop + Sham). The morphology of the stromal layer cells was improved, the cells were slightly activated, and there was no obvious abnormality in nerve reflexes. This indicates that the signs of inflammation in the corneal stromal layer were significantly reduced (Scop + Acup). Quiet stromal cells and nerve fibers (Scop + Flu). The image size is 400 × 400 microns.

As the New Zealand rabbit is used as an animal model, it is difficult to observe the corneal epithelium, which is mainly seen in the IVCM image of the corneal stromal layer (Figure 4). Compared with the other groups, the group Scop and the group Scop + Sham showed a large number of globular immune cells and activated stromal layer cells with unclear borders and irregular sizes. Areas with irregular intercellular spaces were seen. A change in nerve width was seen with the decreasing nerve branch reflectivity. The branches were intermittent, which indicates a state of inflammation Compared with the group Scop, the group Scop + Acup showed that the morphology of the stromal layer cells was improved, the cells were slightly activated, and there was no obvious abnormality in nerve reflexes. This indicates that the signs of inflammation in the corneal stromal layer were significantly reduced. Compared with the group Scop, the cell shape and size of the group Scop + Flu gradually became regular.

### 4.4. Acupuncture Treatment Modulates Corneal Cytokines

**Figure 5:**
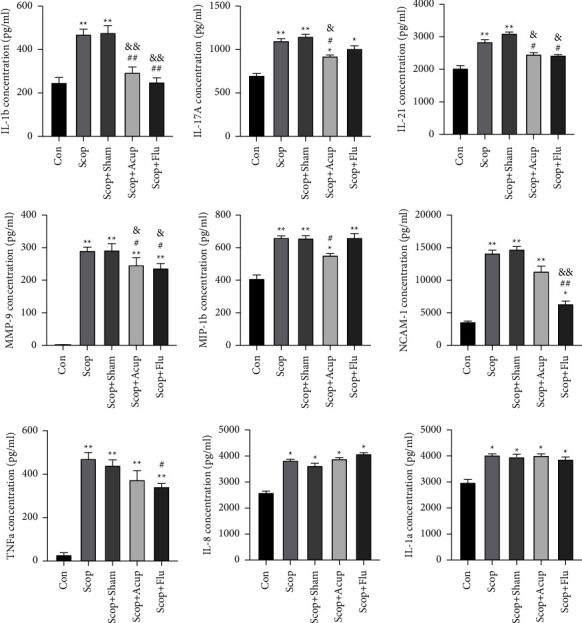
Changes in the cytokines and the chemokines in rabbit corneal on day 35. Quantitative data are expressed as mean ± SEM. Acupuncture stimulus significantly decreased the levels of IL-1b, MMP-9, IL-17A, and IL-21 when compared with the Scop group. The Scop + Flu group significantly decreased the levels of IL-1b, MMP-9, IL-21, MIP-1b, NCAM-1, and TNF-a when compared with the Scop group. ^*∗*^*P* < 0.05, ^*∗∗*^*P* < 0.01 vs. group Con; ^#^*P* < 0.05, ^##^*P* < 0.01 vs. group Scop; ^&^*P* < 0.05, ^&&^*P* < 0.01 vs. group Scop + Sham.

Protein chip technology was used to evaluate the NCAM-1 expression levels in the cornea tissue of IL-1a, IL-1b, IL-8, IL-17A, IL-21, MMP-9, MIP-1b, and TNF-a to study the inhibitory effect of the acupuncture stimulation on the inflammatory response induced by proinflammatory factors and chemokines (Figure 5). Compared with the group Con, IL-8 (*P* < 0.05), IL-1a (*P* < 0.05), IL-1b (*P* < 0.01), IL-17A (*P* < 0.01), leptin (*P* < 0.01), MIP-1b (*P* < 0.01), NCAM-1 (*P* < 0.01), TNF-a (*P* < 0.01), IL-21 (*P* < 0.01), and MMP-9 (*P* < 0.01) were significantly increased in the group Scop and the group Scop + Sham. Compared with the group Scop, the expression of IL-1b (*P* < 0.01), IL-21 (*P* < 0.05), IL-17A (*P* < 0.05), MMP-9 (*P* < 0.05), and MIP-1b (*P* < 0.05) were reduced in the group Scop + Acup. Additionally, IL-1b (*P* < 0.01), IL-21 (*P* < 0.05), NCAM-1 (*P* < 0.01), MMP-9 (*P* < 0.05), and TNF-a (*P* < 0.05) were downregulated in the group Scop + Flu.

### 4.5. Acupuncture Inhibitory Effect on the Corneal NF-*κ*B Is Dependent on *α*7nAChR

**Figure 6:**
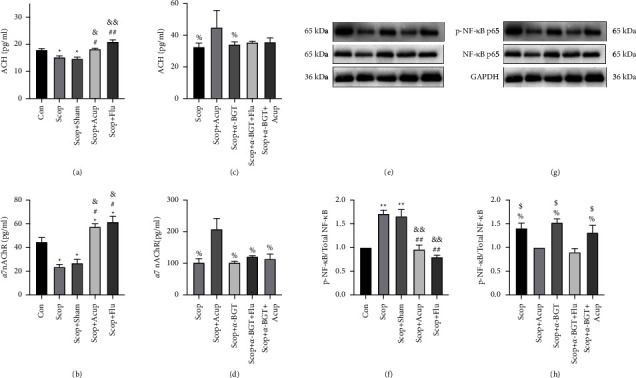
The influence of acupuncture on the changes of corneal ACh and *α*7nAChR was analyzed by ELISA. (a and c) The expression of ACh in each group of corneas and (b and d) the *α*7nAChR expression in each group of corneas. Western blot analysis of the effect of acupuncture on the activation of NF-*κ*B, with GAPDH as control (e–h). Quantitative data are expressed as mean ± SEM. ^*∗*^*P* < 0.05, ^*∗∗*^*P* < 0.01 vs. group Con; ^#^*P* < 0.05, ^##^*P* < 0.01 vs. group Scop; ^&^*P* < 0.05, ^&&^*P* < 0.01 vs. group Scop + Sham; %*P* < 0.05 vs. group Scop + Acup; $*P* < 0.05 vs. group Scop + *α*-BGT + Flu.

The *α*7nAChR antagonist *α*-BGT was subjected to ELISA to detect the contents of ACh and *α*7nAChR and western blot to detect the expression levels of NF-*κ*B p65 and p-NF-*κ*B p65 to verify whether the acupuncture regulated *α*7nAChR and participated in the inhibitory effect of the NF-*κ*B. Compared with the group Con, in the group Scop and the group Scop + Sham the content of ACh (*P* < 0.05) and *α*7nAChR (*P* < 0.01, Figures 6(a) and 6(b)) was significantly reduced, but the expression level of p-NF-*κ*B p65 (*P* < 0.05, Figures 6(e) and 6(f)) increased. Compared with the group Scop, the acupuncture stimulation upregulated the expression levels of ACh (*P* < 0.05) and *α*7nAChR (*P* < 0.01, Figures 6(a) and 6(b)). Compared with that in the group Scop + Acup, the expression of ACh (*P* > 0.05) in the group Scop + Acup + *α*-BGT and the expression of *α*7nAChR (*P* < 0.05) in the group Scop + Acup + *α*-BGT were downregulated (Figures 6(c) and 6(d)), while the expression of p-NF-*κ*B p65 was upregulated (*P* < 0.05, Figures 6(g) and 6(h)). Therefore, acupuncture could increase the expression levels of ACh and *α*7nAChR in the corneal tissue of dry eyes. The deficiency of *α*7nAChR induced by *α*-BGT reversed the inhibition of the NF-*κ*B phosphorylation by the acupuncture stimulus. The Flu, a corticosteroid, inhibited the NF-*κ*B inflammation. The use of *α*-BGT in this trial did not affect the inhibitory effect of fluorometholone on NF-*κ*B. These results suggested that the inhibition of the NF-*κ*B by the acupuncture stimulus was dependent on *α*7nAChR.

### 4.6. Acupuncture Stimulation Regulates Corneal Cytokines through *α*7nAChR

**Figure 7:**
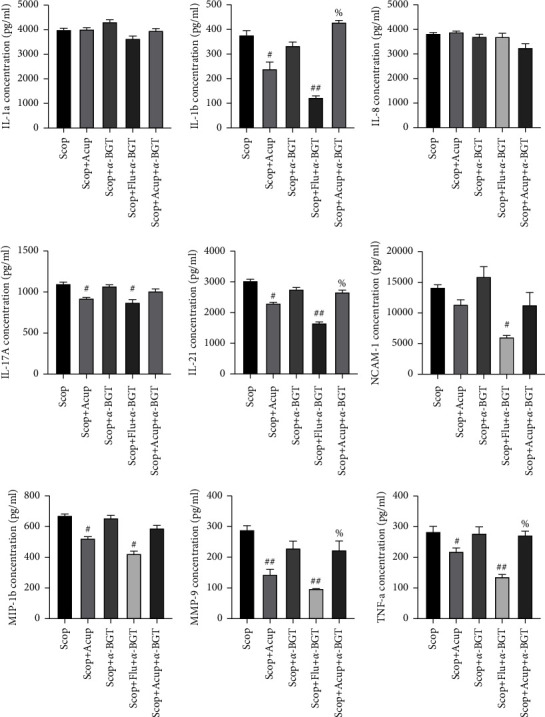
Dependence of the regulation of the acupuncture stimulation corneal cytokines through *α*7nAChR. The *α*7nAChR antagonist *α*-BGT reverses the inhibitory effect of acupuncture stimulation on corneal cytokines. Quantitative data are expressed as mean ± SEM. The group Scop + Acup + *α*-BGT had upregulated IL-1b, IL-21, MMP-9, and TNF-a expression levels when compared with the group Scop + Acup. ^#^*P* < 0.05 and ^##^*P* < 0.01 vs. group Scop; %*P* < 0.05 vs. group Scop + Acup.

After using the *α*7nAChR antagonist *α*-BGT to observe whether acupuncture regulated corneal cytokines by using *α*7nAChR, the protein chip technology was used to continue the evaluation expression levels of IL-1a, IL-1b, IL-8, IL-17A, IL-21, MMP-9, MIP-1b, NCAM-1 and TNF-a in the cornea tissue. Results showed that  compared with the group Scop + Acup, the group Scop + Acup + *α*-BGT had upregulated IL-1b (*P* < 0.05), IL-21 (*P* < 0.05), MMP-9 (*P* < 0.05), and TNF-a (*P* < 0.05) expression levels (Figure 7). This result showed that acupuncture stimulation regulated corneal cytokines through *α*7nAChR.

## 5. Discussion

Acupuncture has a history of more than 2000 years as a means of treatment in traditional Chinese medicine. The therapeutic effects of acupuncture have been recognized and applied in 183 countries [17]. Nowadays, acupuncture is widely used to treat many ophthalmic diseases, such as glaucoma, ophthalmoplegia, nystagmus, and DEDs [18–21]. DEDs are common worldwide. According to the survey carried out by the Tear Film and Ocular Surface Society DEDs Workshop II Epidemiology subcommittee, the prevalence rate of DEDs is around 5% to 50%. Artificial tears are recommended as the first line of treatment for DEDs [21]. Moreover, anti-inflammatory drugs and physical therapy are also used. A large number of clinical reports state that acupuncture can be used for the treatment of DEDs [9, 22, 23]. Interestingly, clinical studies have shown that the effect of acupuncture treatment is not reduced by the use of artificial tears. Acupuncture can improve midterm outcomes for patients [24], so we can speculate that acupuncture be used as an effective complementary therapy to the conventional clinical treatments of DEDs. According to the literature, acupuncture has anti-inflammatory and neuroprotective effects [25]. We used the rabbit DEDs model induced by scopolamine hydrobromide as the observation object. This study found the presence of corneal epithelial damage, tear reduction, shortened tear film rupture time, inflammatory cell infiltration in corneal epithelial and squamous epithelial metaplasia, and corneal cells activated in the stromal layer. The abnormal corneal nerve morphology is in line with previous studies [26–28] and has a strong similarity with the inflammation and damage caused by human DEDs [15]. In the current study, we provided new evidence of acupuncture treatment for DEDs, indicating that acupuncture can reduce the severity of corneal damage in rabbits and can inhibit the formation of DEDs.

Our study demonstrated that the treatment of DEDs with acupuncture may be mediated by the downregulation of proinflammatory factors and chemokines. Inflammation plays a key role in the progress of DEDs. Damage in the ocular surface can cause activation of NF-*κ*B and other related pathways [29], leading to the production of inflammatory cytokines and chemokines, such as IL-1, IL-8, TNF-*α*, IL-17A, and MMP-9 [2, 30, 31]. Inflammatory factors and chemokines could aggravate and amplify the inflammation through their interaction. For example, the degree of corneal epithelial damage is related to the expression of IL-1. IL-1 can induce the expression of several inflammatory cytokines and chemokines [32, 33]. Our data show similar results to previous studies [2, 30, 31]. The concentration of IL-1, IL-8, TNF-*α*, IL-17A, and MMP-9 in the cornea in the DEDs rabbit model were all increased. In addition, we also detected IL -21, leptin, MIP-1b, and NCAM-1 expression, and found they were all increased to varying degrees. Paiva et al. [34] indicated that IL-21 and mRNA transcription levels of the cornea in CD25KO DEDs mice were significantly higher than those in wild mice. Choi et al [35]. detected an increasing MIP-1b expression of the ocular surface in DED patients, especially in Sjogren syndrome (SS) patients; however, no studies of increasing leptin or NCAM-1 in DED patients have been reported. In our present research, we found that acupuncture can improve the expressions of IL-1b, IL-21, IL-17A, and MIP-1b on the cornea in DEDs. This shows that acupuncture can protect the cornea by inhibiting inflammation of the cornea in DED patients.

NF-*κ*B is a transcription factor that cannot be replaced in the inflammatory response. NF-*κ*B can bind to the promoters of a variety of regulatory genes in the inflammatory response, such as IL-1b, IL-6, IL-8, and TNF-*α*. Our data proved that acupuncture can reduce the nuclear metastasis of NF-*κ*B p65, indicating that anti-inflammatory acupuncture can work by inhibiting the NF-*κ*B signaling pathway.

Previous studies have found that CAP is a neural signaling pathway, which relies on the inflammatory reflex of the vagus nerve in the nervous system. It can simultaneously connect the nervous system and the immune system to create an anti-inflammatory effect, releasing ACh and binding to the macrophage surface receptor *α*7nAChR, inhibiting the synthesis of cytokines. In addition, CAP also plays an important role in preventing the release of cytokines [36–38]. Activation of *α*7nAChR in macrophages, monocytes, and other immune cells may downregulate the production of proinflammatory cytokines and attenuate the inflammatory responses by several possible mechanisms: NF-*κ*B activation and JAK-STAT3 pathway [39]. Among these, the key receptor for transmitting cholinergic anti-inflammatory signals is *α*7nAChR. Studies have found that the expression of *α*7nAChR in the lung tissue of acute lung injury rat models is significantly reduced. Pretreatment with electroacupuncture (EA) can prevent the decrease of *α*7nAChR expression and reduce the release of serum and lung inflammatory cytokines after CPB, and the release of protein concentration in BALF and HMGB1. *α*-BGT reduces its beneficial effects [11]. Another study found that EA pretreatment can activate the expression of *α*7nAChR in ischemic brain tissue, thereby reducing the content of iNOS, IL-1*β*, CD86, and proinflammatory cytokines (TNF-*α* and IL-6) and increasing Arg-1, TGF-*β*1, and CD206 and the expression of anti-inflammatory cytokines (IL-4 and IL-10) [12]. Previous experiments have shown that the mechanism of activating *α*7nAChR receptors is through the inhibition of NF-*κ*B [40]. We hypothesized that acupuncture can inhibit the activation of the NF-*κ*B pathway by regulating *α*7nAChR, and thereby the inflammation of the cornea in the DEDs model could be suppressed. Recent studies have reported that electroacupuncture can minimize the damage to the central cholinergic system caused by ischemia, and upregulate the expression of *α*7nAChR in neurons after cerebral ischemia [14]. Consequently, we used scopolamine hydrobromide as a cholinergic inhibitor in this study, which effectively reduced the levels of corneal ACh and *α*7nAChR in the group Scop. After acupuncture treatment, the expressions of corneal ACh and *α*7nAChR were increased. Meanwhile, fluorometholone treatment also upregulated the expressions of corneal ACh and *α*7nAChR. When the *α*7nAChR antagonist *α*-BGT was used, the expression of *α*7nAChR in the mesial cornea of fluorometholone + *α*-BGT was inhibited; however, the NF-*κ*B pathway was still suppressed, suggesting the existence of other anti-inflammatory mechanisms or signaling pathways. The NF-*κ*B pathway of the middle cornea of acupuncture + *α*-BGT was activated. The experimental results prove that acupuncture can inhibit corneal inflammation through the NF-*κ*B pathway and *α*7nAChR regulation. Our study provides a new therapeutic target for DEDs and an important theoretical basis for the anti-inflammatory effect of acupuncture.

## Data Availability

The data used to support the findings of this study can be obtained from the corresponding author upon request.
